# Effects of chronic consumption of specific fruit (berries, cherries and citrus) on cognitive health: a systematic review and meta-analysis of randomised controlled trials

**DOI:** 10.1038/s41430-022-01138-x

**Published:** 2022-04-20

**Authors:** Yueyue Wang, Crystal Haskell-Ramsay, Jose Lara Gallegos, John K. Lodge

**Affiliations:** 1 Department of Applied Sciences, Faculty of Health and Life Sciences, Northumbria University, Newcastle-upon-Tyne, UK; 2 Department of Psychology, Faculty of Health and Life Sciences, Northumbria University, Newcastle-upon-Tyne, UK

**Keywords:** Risk factors, Cognitive control

## Abstract

**Objectives:**

The cognitive-protective effects related to the consumption of a variety of fruits are supported by several intervention studies. This systematic review and meta-analysis compared the magnitude of effects following chronic (≥1 week) consumption of frozen, freeze-dried powder including extracts and juices of fruits, covering berries, cherries and citrus, on cognition and mood in adults.

**Methods:**

PubMed, Web of Science, Scopus, and psycARTICLES were searched from inception until February, 2021. Inclusion criteria were randomised controlled trials assessing memory, executive function, psychomotor speed, mood and mini mental state examination in adult participants ≥18 years of age. Cognition was tested by global or domain specific tasks.

**Results:**

Out of 13,861 articles identified, 16 papers were included; 11 studies provided suitable data for meta-analysis. Fourteen studies reported improvement or trend for improvement in cognition, five studies assessed mood and one study supplementing grape juice found trend for mood improvement. From the meta-analysis, cherry juice supplementation was suggested to improve psychomotor speed by −0.37 of standardised mean difference (95% CI [−0.74, 0.01]) in reaction time (*P* = 0.05).

**Conclusions:**

The meta-analysis did not sufficiently support a role for fruits or fruit forms to improve cognition and mood.

## Introduction

Age-induced cognitive decline is a common and important phenotype that is likely to be associated with increased disease risks such as dementia [1]. Ageing is associated with an increased susceptibility to chronic organ disease and decline of metabolic and immune functions which impact on the brain [1]. Aside from cognitive decline with ageing, an increased age-associated risk of neurodegenerative disorders such as dementia and Alzheimer’s disease is prevalent [2]. Therefore, the measurement and treatment of cognitive impairment is important due to the rising prevalence of dementia [2] and the benefits of early detection and prevention [3].

The rate of age-related cognitive impairment is mainly influenced by lifestyle behaviours, including diet [4, 5]. Fruits and vegetables represent a rich source of antioxidants including vitamins (vitamin C, B complex and E etc.), carotenoids and polyphenols, which have been shown to improve cognition [6–8]. A number of potential underlying mechanisms have been identified including the interaction of fruit polyphenols, carotenoids and vitamins with intracellular neuronal and glial signalling pathways, regulation of cerebral blood flow, and protection against neurotoxins and neuroinflammation [8–10]. A dose-response meta-analysis including nine studies (five cohort studies and four cross-sectional studies) with a total of 31,104 participants suggested approximately a 13% (OR = 0.87, 95% CI 0.77–0.99) reduction in cognitive impairment and dementia risk by an increment of 100 g per day of fruit and vegetable consumption [11]. A number of intervention studies also showed positive results indicating that intake of a range of flavonoid-rich fruit (e.g. blueberry, orange juice) improves both immediate and chronic cognitive performance or mood in older adults [12]. From a nutritional perspective, employing single whole foods instead of single components such as a supplement in interventions is more appropriate since synthetic single nutrient supplements are likely to be metabolised through different pathways to natural bioactive compounds [13].

Fruit can be consumed in a variety of forms following different processing methods, for example as fruit juices, smoothies, frozen fruit, and freeze-dried fruit powders and a recent paper emphasized the need for additional research assessing the effect of processed fruit on health, as fruit in processed forms (e.g. processed powder) provide consumer options whilst also reducing costs and food waste [14]. These different fruit forms could be an effective method to increase overall fruit consumption and it is important to determine the impact of different fruit groups and delivery forms on cognition in order to better inform the public. Therefore, the protective effects on cognition of whole fruit intervention or fruit intervention in different forms (e.g. powder, juice) instead of single molecules, such as polyphenols, are worth exploring.

Inconsistent findings have been reported on fruit interventions for cognitive effects [15, 16]. Whilst small sample size is indicated as a factor in null findings, the length of intervention as well as type and form of fruit may also be important. There could be metabolic difference between acute and chronic polyphenol-rich fruit interventions. The circulating metabolites, including polyphenol metabolites only remained in the cerebral blood flow (CBF) circulation for an approximately 1–2 h peak Cmax in plasma [17–19]. Emerging evidence from acute interventions employing a range of fruit juices and dried powders also indicates that polyphenol-rich fruit-based interventions e.g. citrus, grape, and blackcurrant juice and powder may benefit brain function [20–22].

In terms of nutritional value, fruit juices and dried fruit powders retain polyphenols, vitamins and minerals that are bio-accessible despite fibre and other nutrient losses that occur during processing [23, 24]. Fruit processing, such as smashing or thermal treatment, can damage the cell structure of whole fruit releasing cytoplasmic content that can make bioactive compounds more accessible for absorption [25]. Moreover, the presence of other constituents formed from the technological process or added from food matrix such as sugar could modify the bioavailability of the bioactives due to their ability to bind, solubilize, or stabilize [26]. Research has explored the difference in nutritional value and bioavailability of bioactives between whole fruit and processed fruit. One in vitro gastrointestinal digestion model study showed that mulberry juice still contains 60 - 70% of the anthocyanin content compared to unprocessed raw mulberry [27]. Furthermore, Kuntz et al. compared the bioavailability of selected anthocyanins from grape and blueberry juice with a smoothie and found no difference in plasma pharmacokinetics and recovery of the major anthocyanin species. However, significantly higher concentrations of the phenolic acid 3,4-dihydrobenzoic acid were shown after ingestion of the juice [28].

It is also worth noting that the cognitive domains categorised in intervention studies often vary depending on the cognitive ability that researchers intend to assess with different tasks applied [26]. Therefore, the impact of fruit interventions on specific cognitive domains cannot be quantitatively compared because of the large variability in the assessment tools and scoring interpretations. This concern has also been highlighted in other dietary trials assessing cognition [29]. Here, we have systematically reviewed and meta-analysed available fruit interventions to evaluate the chronic effects (≥1 week) of whole, powdered, and juiced fruit, specifically anthocyanin-rich berries and cherries, and flavonoid-rich citrus fruit on cognition in randomised controlled trials (RCTs). We also categorised specific tasks, depending on the assessed cognitive ability/domain for each RCT, in order to achieve quantitative comparison across RCTs. Due to the impact of mood on cognition, we also included assessment of mood outcomes here [30].

## Methods

### Study eligibility

We searched for studies investigating the effect of berry, cherry and citrus fruit supplementations on cognition and mood. Berries so defined includes grapes, blueberries, strawberries etc; citrus includes oranges etc; cherry is categorised as stone fruit [31]. The following specific inclusion criteria were applied: (1) study design: RCTs; (2) subjects: adult subjects ≥18 years of age; (3) interventions: only chronic intervention studies for at least one week providing or promoting citrus, cherry, or berries including blueberry, grape, blackberry, raspberry and cranberry in various forms (e.g. their juices) to be consumed without acute supplementation prior to testing; (4) control: control groups without components of citrus, cherry, or berries, likely isoenergetic placebo group; (5) outcomes: cognitive function and mood (described below); (6) Only English-language and peer-reviewed articles were included. No restriction of publication year was applied.

### Data sources

This review is in line with the PICOS (population, intervention, comparator, outcome, study design) framework (Supplemental Table 1). The systematic review was conducted with a prospective protocol in accordance with Cochrane Handbook for Systematic Reviews of Interventions version 5.1 [32] and Centre for Reviews and Dissemination Guidelines [33] and was reported according to PRISMA guidelines [34] (Supplemental Table 2). The protocol was registered with PROSPERO, the International Prospective Register of Systematic Reviews (Registration number CRD42018091896). This protocol includes the investigation of the impact of these fruits on the risk factors of cardiovascular diseases, however the analysis has been reported elsewhere [35]. The search started from inception until February 2021 using PubMed, Web of Science, Scopus and psycARTICLES. The search strategy was as following: (Fruit OR citrus OR orange OR berry OR berries OR grape OR blueberry OR blueberries OR blackberry OR strawberry OR strawberries OR blackcurrant OR blackberries OR raspberry OR raspberries OR cranberry OR cranberries OR cherry OR cherries) AND (cogniti* OR memory OR “executive function” OR “reaction time” OR “psychomotor speed” OR attention OR mood) AND (trial OR intervention). Full electronic search strategy for PubMed was added in Supplemental Table 3.

### Study selection

Two researchers (YW and JLG) assessed articles independently for inclusion eligibility. All records were exported to EndNote X8 reference management software. Articles were moved to the next screening phase or discarded when full disagreement was reached. Any disagreements that were not resolved were handled by CHR and JKL serving as arbitrators. No disagreements occurred during the selection phase. The selection of eligible studies was based on 2 steps. Firstly, the title and abstract of each study was screened for relevance; full texts were then reviewed for those with potential for inclusion. Reference lists of included papers and relevant systematic reviews were also supplemented by hand-searching for additional articles.

### Data abstraction

Data were extracted by YW and JLG independent of each other, their selections for accuracy were reviewed in meeting. Corresponding authors were contacted via e-mail for requested information if there was missing data or for clarification. A pre-defined data extraction form was used to input study data, which includes information on (1) author and published year; (2) study design; (3) population characteristics (ethnicity, mean age, sex, mean BMI, health status and sample size at baseline); (4) treatment details (intervention type, length, dosage and frequency); (5) control group settings; (6) retention rate; (7) measured cognitive testing scores for both experimental group and placebo group at baseline and the longest post-intervention time point to avoid the bias of selectively choosing data (if applicable); (8) recording any data adjustments made for physical activity level among the included studies. Primary outcomes of the analyses were cognitive and mood scores after intervention and placebo treatment. The cognitive function measured in each study was categorized into memory, executive function and psychomotor speed domains for meta-analysis. The domain categorization in this review was based on a commonly used approach to understand cognitive domains [36].

### Risk of bias and quality assessment

Study quality was assessed by Cochrane Risk of Bias (RoB2) tool with assessment of five components, D1 of randomisation process, D2 of deviations from intended interventions, D3 of missing outcome data, D4 of measurement of the outcome and D5 of the selection of the reported results [29]. The overall risk of study bias was rated by low risk, some concerns or high risk. Grading of Recommendations Assessment, Development, and Evaluation (GRADE) evidence profile [37] was also applied to evaluate the risk of bias, inconsistency, indirectness, and imprecision of evidence in the aspects of assessed executive function, memory, psychomotor speed and mood in the RCTs included in the present review. Publication bias was also assessed by Funnel plot and Egger’s test where available [38].

### Data synthesis

R studio version 3.5.2 [39] and the package “meta” [40] were used to pool and meta-analyse data from collected studies. Means and SDs from endpoint and baseline data for intervention and control group were obtained from long-term (≥1 week) studies. All pooled results were presented as weighted mean difference or standardised mean difference with 2-sided *P* values and 95% of confidence intervals (CIs) in forest plots. Sensitivity analysis was performed to investigate the impact of studies adjusting for participants’ physical level and also the impact of juice quality on the meta-analysis results [41, 42]. However, insufficient study data was obtained to implement subgroup analysis to further explore the impact of the variations induced by study design and participant characteristics.

As shown in Table 1, based on the categorised cognitive tasks, meta-analyses investigating the effects of berry interventions on memory, executive function and psychomotor speed [43–49] and 2 cherry juice studies assessing executive function and psychomotor speed were carried out [41, 50]. Two grape powder studies assessing MMSE (Mini Mental State Examination), which measures cognitive impairment, were also included [51, 52]. Memory and executive function both encompass the essential cognitive processes in a person’s life [53]. Psychomotor speed assesses the individual’s ability to detect and respond to a stimulus and therefore reflects the relationship between cognition and physical movements [54].

**Table 1:** Cognitive domain classification under each intervention type.

Cognitive domain	Intervention type	Study	Cognitive task entered into meta-analysis	Unit of test scores	Results of the chosen task^a^
Memory	grape juice	Lamport, et al., [43] (UK)	Visual verbal learning test-Immediate recall	accuracy/%	No changes
	cranberry juice	Crews et al., [44] (US)	Delayed free recall	correct numbers	No changes
	blueberry powder	Miller, et al., [48] (US)	CVLT list A 1 free recall	correct numbers	No changes
Executive function	grape juice	Lamport, et al., [43] (UK)	RVIP correct	correct responses	No changes
	cranberry juice	Crews et al., [44] (US)	Digit symbol	raw score	No changes
	blueberry powder	Boespflug et al., [46] (US)	2-Back task	arc	No changes
	blueberry juice	Bowtell et al., [45] (UK)	2-Back task	arc	No changes
	cherry juice	Chai, S. C., et al., [41] (US)	Digit span	score	No changes
		Kent, et al., [50] (Australia)	Digit span backwards task (short-term memory)	score	Final score between groups (*P* = 0.02)
	Aronia melanocarpa extract powder	Ahles et al., [49] (Netherlands)	The number cross-out task	Correct responses	No changes
Psychomotor speed	grape juice	Lamport, et al., [43] (UK)	RVIP reaction time	ms	No changes
	cranberry juice	Crews et al., [44] (US)	Trial making task reaction time	total time	No changes
	frozen blueberry	Schrager, et al., [47] (US)	Simple reaction time	ms	No changes
	blueberry powder	Boespflug et al., [46] (US)	2-Back task reaction time ms	ms	No changes
	cherry juice	Kent, et al., [50] (Australia)	Trail making task reaction time	ms	No changes
		Chai. S. C., et al., [41] (US)	RTI reaction time	ms	No changes
MMSE	grape powder	Calapai, et al., [51] (Italy)	MMSE	score	Change score between groups (*P* < 0.01)
		Lee, et al., [52] (US)	MMSE	score	No changes

*arc* Arcsine transformation of the square root of the proportion of correct answers, *CVLT* California Verbal Learning Test, *RVIP* Rapid Visual Information Processing, *RTI* Reaction Time Test, *MMSE* Mini Mental State Examination.

^a^All summarised results were compared to the control group.

Statistical heterogeneity was estimated by Cochrane *Q* statistics and the consistency of study results was assessed by *I*^*2*^ statistics as an extension of Cochrane *Q* statistics, which depicts the proportion of the variability in treatment effect rather than sampling error (chance) that accounts for the real study differences (heterogeneity) and an *I*^*2*^ > 50% was considered for high heterogeneity level [55].

Standard deviation of mean difference (SMD) was used for studies assessing MMSE, memory, executive function, and psychomotor speed, where studies used different assessments or different measurement units. Variance of treatment effects across studies due to real treatment differences and/or sampling variability (chance) was assumed by a Hartung-Knapp-Sidik-Jonkman random-effects meta-analysis model [56].

## Results

### Literature search

**Fig. 1:**
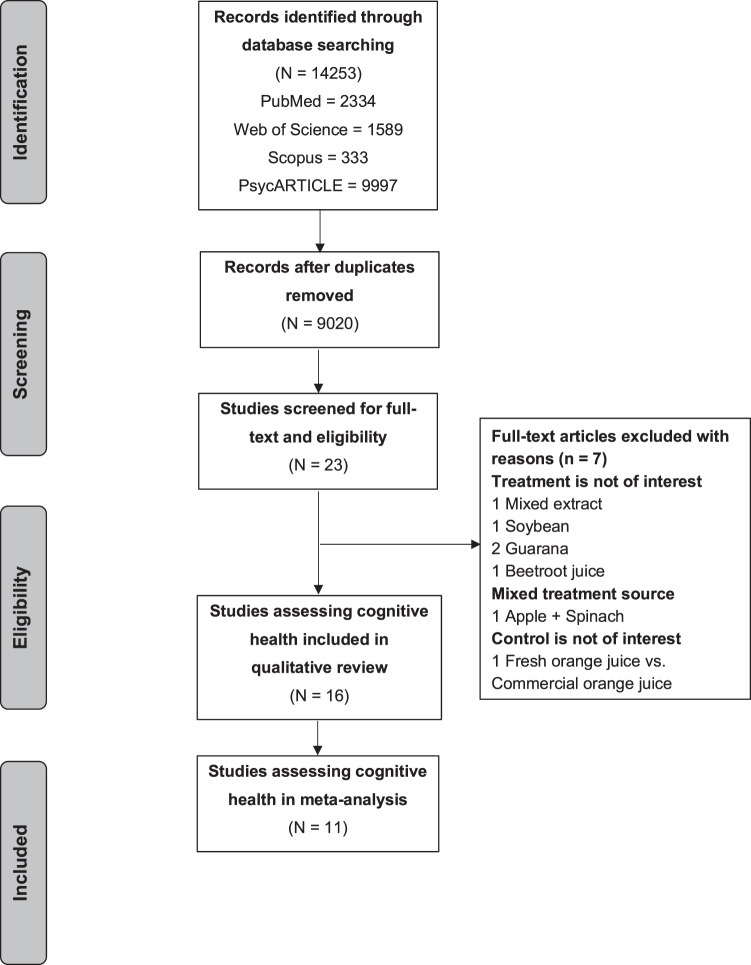
Flow diagram of study selection.

In accordance with PRISMA guidelines [34], the selection process for included studies is shown in Fig. 1. The initial search produced 14,253 articles from the four databases, no additional article was added from manual search through reference lists of articles previously identified. This record was reduced to 9020 articles after duplicates were removed. After screening of titles and abstracts for eligibility, 8997 articles were excluded either due to not being human intervention studies or abstract only. The final selection identified 23 trials assessing cognition, seven articles were further excluded after checking full-text eligibility. Sixteen trials were included in this review, 11 trials from these were included in the meta-analysis.

### Study characteristics

Among 16 included studies in this review (Table 2), there were 3 crossover RCTs [43, 57, 58] and 13 parallel RCTs [41, 44–52, 59–61]. The average age of the participants in the interventional and control group for included studies were 65.49 ± 15.78 and 65.26 ± 15.61 years old respectively. Thirteen of these studies recruited older participants (aged 60 years or older) [41, 44–48, 50–52, 57, 59–61]. Baseline characteristics of participants varied across interventions, 10 studies included healthy participants at baseline [41, 43–45, 47, 49, 51, 57, 58, 61], five studies included older people manifesting cognitive decline [46, 48, 52, 59, 60] and the remaining one study recruited diagnosed mild to moderate dementia [50].

**Table 2:** GRADE evidence profile.

Certainty assessment						No of patients					Certainty	Importance
No of studies	Study design	Risk of bias	Inconsistency	Indirectness	Imprecision	Other considerations	Berries, cherries and oranges supplemented randomised controlled trials	Placebo controls	Relative (95% CI)	Absolute (95% CI)		
Executive function (follow up: range 8 weeks to 16 weeks)												
6	RCT	serious^a^	not serious	not serious	not serious		134	135	3	mean 0.08 SMD higher (1.05 lower to 1.08 higher)	3	IMPORTANT
Memory (follow up: range 5 weeks to 24 weeks)												
5	RCT	serious^b^	not serious	not serious	not serious		156	151	3	mean 0.02 SMD higher (1.04 lower to 0.92 higher)	3	IMPORTANT
Psychomotor speed (follow up: range 5 weeks to 16 weeks)												
7	RCT	serious^c^	not serious	not serious	not serious		203	189	3	mean 0.27 SMD higher (1.08 lower to 2.88 higher)	3	IMPORTANT
Mood (follow up: range 1 week to 6 months)												
10	RCT	not serious	not serious	not serious	not serious		232	225	3	mean 24.9 % lower (0 to 0)	3	IMPORTANT

Author(s):

Question: Berries, cherries and oranges supplemented randomised controlled trials (RCT) compared to placebo controls for improving cognitive health

*CI* Confidence interval.

Explanations:

^a^(Boespflug et al., [46]) study did not specify randomisation and blinding process.

^b^(Chai et al., [41]) study was not blinded.

^c^(Boespflug et al., [46]) and (Chai et al., [41]) studies presented high risk of bias due to aforementioned reasons.

There were 9 studies supplementing fruit juice and concentrate [41, 43–45, 50, 57–60], six studies supplemented fruit powder [46, 48, 49, 51, 52, 61] and only one study used whole frozen fruit [47]. Among 16 studies, the mean intervention duration was 13 weeks, ranging from 1 week to 6 months. Two studies supplementing Aronia melanocarpa berry and grape powder used placebo powder composed of maltodextrin as control [49, 62]. One blueberry concentrate intervention used isoenergetic synthetic blackcurrant and apple cordial as control [45]; the orange juice intervention used equicaloric low-flavanone (37 mg) orange-flavoured cordial as control [63]; one cherry juice intervention used flavonoid-devoid apple juice as control [50]; the frozen blueberry intervention used carrot juice with low anthocyanins as control [47]. The rest of the studies used placebo beverage or powder matched for energy, carbohydrate, flavour but devoid of polyphenol content as a control [41, 43, 44, 46, 58–60, 64, 48].

Among the juice supplementations, three studies were accumulated under the groupings of grape juice with mean dosage of 408 ml/d containing173.4 mg, 173.4 mg and 167 mg anthocyanins respectively [43, 59, 60]; three were cherry juice including concentrate with mean dosage of 247 ml/d containing 138 mg anthocyanins, 320 mg anthocyanins, and 450 mg total polyphenols respectively [41, 50, 58], one was cranberry juice with 32 ounces/d (around 942 ml) containing 435 mg total polyphenols [44]. One was blueberry juice concentrate with 30 ml/d [45] containing 387 mg anthocyanidins and one was orange juice study with 500 ml/d containing 305 mg flavanones [57]. Three studies supplemented blueberry powder with mean dosage of 16.3 g/d containing 363 mg anthocyanins, 460.8 mg anthocyanins, and 70 mg total polyphenols respectively [46, 48, 64] and two supplemented grape powder with mean dosage of 48.5 g/d containing 22.3 mg and 12.5 mg anthocyanins respectively [52, 62]; portion conversion of powder to whole fruits were provided in three studies [46, 48, 52]. For example, Lee et al. supplemented grape powder that was comparable to three servings of fresh grapes daily (approx. 504 g/d fresh grapes) [52]. Two studies supplementing blueberry powder were equivalent to providing one cup and 1.5 cups of fresh blueberries as measured in FDA recommended fruit portions respectively [46, 48]. Ahles et al. supplemented 90 mg and 150 mg Aronia melanocarpa berry extract powder providing 16 mg and 27 mg anthocyanins respectively without indicating portions of equivalent whole berry [49]. Schrager et al. supplemented 200 g/d whole frozen blueberries without measuring total polyphenol or anthocyanins levels [47].

### Study quality

**Fig. 2:**
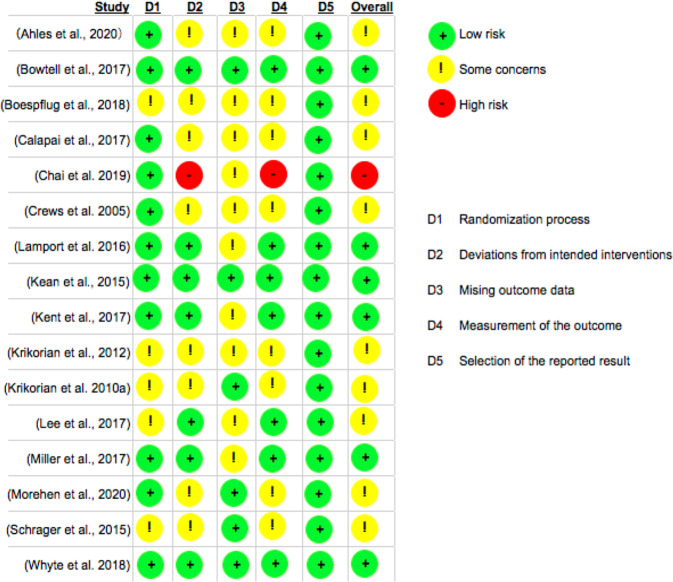
RoB2 assessment of study quality. RoB2: A revised Cochrane risk-of-bias tool for randomized trials.

This review assessed RCTs’ quality via RoB2. Figure 2 presents the RoB2 assessment for each study. Eleven studies presented low risk in the evaluation of randomisation process and five studies presented some concerns. Seven studies presented low risk in the evaluation of deviations from intended interventions, eight studies presented some concerns, and one study presented high risk. Six studies presented low risk in the evaluation of missing outcome data, 10 studies presented some concerns. Seven studies presented low risk in the measurement of the outcome, eight studies presented some concerns and one study presented high risk. All 16 studies presented low risk in the selection of the reported result. Six studies presented low risk for overall study risk of bias, nine studies presented some concerns for overall study risk of bias and one study presented high risk for overall study risk of bias.

As summarised in Table 2 of GRADE evidence, there may be serious risk of bias in the assessment of executive function, memory and psychomotor speed in the RCTs included in this review. The risk of bias in the assessment of mood and the risk of inconsistency, indirectness, or imprecision of the assessment of executive function, memory, psychomotor speed and mood was not serious in the RCTs included in this review.

### Findings from the studies included in the systematic review

As shown in Table 3, all 16 included studies have assessed cognition with 14 studies reporting improvement or trend for improvement in cognition; 10 studies also assessed mood and one study supplementing grape powder found trend for mood improvement. The largest portion of studies supplemented frozen, juiced or powdered berries (12 out of 16 studies) with blueberries (*n* = 5) and grapes (*n* = 5) being the most intensively studied interventions.

**Table 3:** Summary of interventions assessing the effect of fruit supplementation on cognition and mood.

Reference	Study design	Sample (sample at baseline/N, age (years), male (%), mean BMI (kg/m2), health status)	Intervention type, dose and duration	Cognitive tasks	Effects of intervention	Overall risk of bias
Ahles et al. [49]	Double-blind, placebo-controlled, parallel RCT	*N* = 35; 53 ± 1 years old; male 32%; BMI 29.5 ± 0.4; healthy	90 mg Aronia melanocarpa, a berry extract (AME) capsule consisted of 16 mg anthocyanins; 150 mg AME consisted of 27 mg anthocyanins; Maltodextrin containing capsules (150 mg) were used as placebo; 24 weeks	Pegboard dominant hand score; Pegboard non-dominant hand score; Total correct—total incorrect; Total edited—total incorrect and missed; Stroop Interference; Mood (T-Scores); BDNF	AME improved psychomotor speed compared to placebo (90 mg AME: change = −3.37; *P* = 0.009). Attention, cognitive flexibility, BDNF, and mood were not affected.	Moderate
Bowtell et al. [45]	Double-blind, parallel-group RCT	*N* = 12; 67.5 ± 3.0 years old, male 58.33%; BMI 25.9 ± 3.3;	30 ml blueberry (BB) concentrate (blueberry active) providing 387 mg anthocyanidins, 12 weeks, isoenergetic synthetic blackcurrant and apple cordial as control	Detection Task, Groton maze timed chase test and learning test, sequential letter 1-back and 2-back tasks, fMRI, serum sample	Improved executive function (Groton maze learning task accuracy). Also improved task-related brain activation (Brodman areas, presumes, anterior cingulate, insula and thalamus regions)	Low
Boespflug et al. [46]	Double-blind, parallel-group RCT	*N* = 8; 75.5 ± 4.8 years; male 37.5%; BMI 26.2 ± 3.6; age-related memory decline.	Blueberry powder: 12.5*2 g equivalent to 148 g whole blueberry, 16 weeks, placebo powder: 12*2 g	Sequential letter n-back tasks, fMRI	Trend for improving working memory at larger sample size (effect size *d* = 1.02). Also increased BOLD activation in the left pre-central gyrus, left middle frontal gyrus, and left inferior parietal lobe during tasks after BB treatment.	Moderate
Calapai et al. [51]	Parallel RCT	*N* = 57; 56–75 years; male 48.2%;BMI 23.2 ± 1.0; healthy.	250 mg/d Cognigrape-V. vinifera fruit extracted powder and maltodextrin (30–40%), 12 weeks, placebo was composed of maltodextrin	MMSE score, BDI, HARS, RBANS	Improved attention, language, immediate and delayed memory. Supplementation also produced a significant reduction in BDI ( − 15.8%) and HARS ( − 24.9%) scores with respect to baseline levels (p < 0.0001) and placebo (p < 0.0001 for BDI and p < 0.05 for HARS.	Moderate
Chai et al. [41]	Parallel RCT	*N* = 17; 70.0 ± 3.7 years; male 40%; BMI 28.5 ± 3.7; older adults with normal cognitive function	480 ml tart cherry juice (68 ml Montmorency tart cherry juice concentrate was diluted with 412 ml water) per day for 12 weeks; placebo consisted of mixing unsweetened black cherry flavoured Kool-Aid (Kraft Foods, United States) with water. Dextrose and fructose were added to match the carbohydrate content found in tart cherry	Memory ability, Memory contentment, Memory strategy, digit span, PAL first trial memory, PAL total errors adjusted, RTI movement time, RTI reaction time, RVIP A, RVIP mean latency, SWM strategy, SWM total error	Increased subjective memory in the domain of contentment with memory by 5% and reduced movement time by 4% in comparison with the control drink. Also reduced errors in episodic visual memory by 23% compared to control drink as assessed by PAL task.	High
Crews et al. [44]	Double-blind, parallel-group RCT	*N* = 24: 69.28 ± 6.45 years; male 42%; BMI N/A; healthy.	909 ml/d 27% cranberry juice for five weeks, placebo drink	Immediate free recall, long term storage, short-term recall, long-term retrieval, consistent long-term retrieval, random long-term retrieval, cued recall, delayed free recall, delayed recognition, Faces I, Faces II, Digit symbol, Part A, Part B, Word page, colour page, colour-word page	A nonsignificant trend (*P* = 0.123) observed for twice as many subjects of subjective, self-report improved memory in cranberry group compared to placebo controls.	Moderate
Lamport et al. [43]	Double-blind, randomized crossover design	*N* = 25; 43.2 ± 0.6 years; male 0%; BMI 24.6 ± 0.5; healthy.	355 ml/d concord grape juice, 6 weeks and 12 weeks, energy-, taste-, and appearance-matched placebo	VVLT and VSLT IR & DR, RVIP, Grooved Pegboard, Tower of Hanoi, SBP, DBP, Subjective Mood, Driving performance	Better immediate spatial memory and aspects of driving performance after GJ intake. No difference in mood between groups	Low
Kean et al. [57]	Double-blind, randomized, crossover	*N* = 37; 66.7 ± 5.3 years; male 35.13%; BMI 26.1 ± 1.1; healthy.	High-flavanone (305 mg) 100% orange juice, 500 ml/d for 8 weeks, equicaloric low-flavanone (37 mg) orange-flavoured cordial (500 mL) as control	SBP, DBP, DSST, DSST dual, Go-NoGo RT, LF, LM, Serial Sevens, CERAD Immediate and Delayed, SWM, Immediate and Delayed VPA, PANAS Positive and Negative Affect Scale	Improved cognition (significant drink x visit interaction for global cognitive function and executive function). No effect on mood and BP.	Low
Kent et al. [50]	Parallel RCT	*N* = 24; 78.9 ± 5.2 years; male 51%; BMI 25.7 ± 3.4; mild to moderate dementia.	200 ml/d cherry juice, 12 weeks, flavonoid-devoid apple juice as control	RAVLT, SPOT, Boston naming test, TMT, Digit Span Backwards Task, Category and Letter Verbal Fluency, SBP, DBP, serum sample	Improved cognition in memory and executive function and reduced SBP of 7.7 mmHg after juice treatment. No effect on Vitamin C and inflammatory markers.	Low
Krikorian et al. [59]	Parallel double-blind RCT	*N* = 11; 76.9 ± 6.1 years; male 52.38%; BMI N/A; mild cognitive impairment	100% Concord grape juice 6.3–7.8 mL/ kg/d, 3 portions daily, 16 weeks, placebo beverage.	CVLT, GDS, fMRI, SBP, DBP	Attenuated cognitive error (5.03 vs 7.16 interference errors on recognition memory task) and great activation in right superior parietal cortex and right middle frontal cortex regions after juice treatment. No effect on mood and BP.	Moderate
Krikorian et al. [60]	Parallel double-blind RCT	*N* = 5; 78.2 ± 5.0 years; male 66.67%; BMI N/A; older adults with early memory decline but not dementia.	100% Concord grape juice, 6 and 9 ml/kg/d, 3 portions daily, 12 weeks, placebo beverage.	CVLT, SPALT, GDS, glucose and insulin	Improved memory and insulin level after juice treatment. No effect on mood.	Moderate
Lee et al. [52]	Parallel double-blind placebo controlled RCT	*N* = 5; 72.2 ± 4.7 years; male 50%; BMI N/A mild cognitive decline.	Grape formulation ---freeze-dried grape powder in 8 oz. water, 72 g/d (3 standard servings daily), 6 months, placebo formulation matched in appearance, flavour, smell, volume and content of fructose and glucose but free of polyphenols	ADAS-Cog, MMSE, Hopkins Verbal Learning Test-Revised, Benton Visual Retention Test, Rey-Osterreith CFT, Boston Naming Test, LF, Category Fluency, Stroop, TMT Parts A and B, Wisconsin Card Sorting Test-64, WAIS-III Tasks, Wechsler Test of Adult Reading, Memory Functioning Questionnaire, Hamilton Mood Scales, Neuroimaging sVOI	Attenuated decline in brain metabolites at regions of right posterior cingulate cortex and left superior posterolateral temporal cortex and improved correlated attention after grape treatment. No effect on mood.	Moderate
Miller et al. [48]	Parallel double blind placebo-controlled RCT	*N* = 18; 67.8 ± 4.6 years; male 28%; BMI 24.1 ± 3.7; age-related motor and cognitive decline.	24 g/d freeze-dried blueberry powder. 90 days, placebo powder	CVLT, ANT, DS, TMT, TST, wMWM, GDS, POMS	Attenuated cognitive error and improved executive function after blueberry treatment. No effects on mood.	Low
Morehen et al. [58]	Cross over single blind RCT	*N* = 11; 18 ± 1 years; male 100%; BMI 27.83 ± 2.51; professional rugby league players	60 mL cherry concentrate (30 mL*2) in 100 mL water, placebo drink commercially available fruit cordial, mixed with water and maltodextrin, matched for energy and carbohydrate content daily intake for 1 week.	Self-reported subjective wellness including rating of perceived sleep quality, fatigue, muscle soreness, mood and stress using a 1–5 Likert scale.	No significant changes in sleep, fatigue or mood (*P* > 0.05) were observed pre to post-match or between groups	Moderate
Schrager et al. [47]	Parallel RCT	*N* = 13; 69.5 ± 9.3 years; male 45%; BMI 26.4 ± 3.9; healthy	6-cup (48 ounce (1.4 kg))/week frozen blueberries, 6 weeks, placebo/carrot juice with low anthocyanins contents	Grip strength, SRT, adaptive gait tests, TMT-B	Reduced errors (76.9% vs 57.1% of participants in BB vs Control had reduced errors) and improved mobility after BB treatment.	Moderate
Whyte et al. [61]	Parallel double blind placebo-controlled RCT	*N* = 29; 70.8 ± 3.88 years; male 38.50%; BMI 27 ± 4; healthy.	500 mg, 1000 mg blueberry powder or 111 mg purified blueberry extract for 24 weeks, colour matched placebo	Rey’s Auditory Verbal Learning Task (RAVLT), Picture Recognition Task, Corsi Block Task, Stroop Task, and Modified Attention Network Task (MANT), the Serial 3 s, Serial 7 s, and Sternberg task, the PANAS-NOW	No effect on cognition and mood after the blueberry powder intervention. Improved episodic memory performance in delayed word recognition and marginally significant improved visuo-spatial Corsi Block performance at 3, but not 6, months following blueberry extract intervention.	Low

*ADAS* The Alzheimer’s Disease Assessment Scale, *ADCS-ADL* The Alzheimer’s Disease Cooperative Study-Activities of Daily Living, *AG* Affect Grid, *ANT* Attention Network Task, *BDI* Beck Depression Inventory, *CBFV* Cerebral Blood Flow Velocity, *CERAD* Consortium to Establish a Registry for Alzheimer’s Disease, *CFT* Complex Finger Tapping, *CPT* Continuous Performance Task, *CRT* Choice Reaction Time, *CS* Contrast Sensitivity, *CVLT* California Verbal Learning Test, *DBP* Diastolic Blood Pressure, *DPR* Delayed Picture Recognition, *DRS* The Dementia Rating Scale, *DSST* Digit Symbol Substitution Test, *DV* Digit Vigilance, *DVR* Delayed Verbal Recall, *DWR* Delayed Word Recognition, *EEG* Electroencephalogram, *fMRI* Functional Magnetic Resonance Imaging, *FVT* Freiburg Vision Test, *GDS* Geriatric Depression Scale, *HARS* Hamilton Anxiety Rating Scale, *IWR* Immediate Word Recall, *IVR* Immediate Verbal Recall, *LF* Letter Fluency, *LM* Letter Memory, *MMSE* Mini Mental State Examination, *NWM* Numeric Working Memory, *NIRS* Near-IR spectroscopy, *NPI* Neuropsychiatric Inventory, *PANAS* Positive and Negative Affect Scale, *PP* Picture Presentation, *POMS* Profile of Mood States questionnaire, *RAVLT* Rey Auditory Verbal Learning Test, *RBANS* Repeatable Battery for the Assessment of Neuropsychological Status, *RT* Reaction Time, *RVIP* Rapid Visual Information Processing, *SBP* Systolic Blood Pressure, *SFT* Simple Finger Tapping, *SPOT* Self-ordered Pointing Task, *SPALT* Spatial Paired Associate Learning Test, *SRT* Simple Reaction Time, *sVOI* Standardised Volume of Interest, *SWM* Spatial Working Memory, *TMT* Trail Making Task, *TST* Task Switching Task, *VAS* Visual Analogue Scales, *vMWM* Virtual Morris Water Maze, *VPA* Verbal Paired Association, *VSLT* Visual Spatial Learning Test, *VVLT* Visual Verbal Learning Test, *WAIS* Wechsler Adult Intelligence Scale, *WFC* Word Fragment Completion, *WMS* Wechsler Memory Scale, *WP* Word Presentation.

As shown in Table 3, one frozen blueberry study reported improved motility with a large effect size (Cohen’s *d* = 1.03) and reduced step errors (76.9% vs 57.1% of participants in intervention vs control group) with a large effect size (Cohen’s *d* = 1.16) [47]. Ahles et al. found improved psychomotor speed after supplementing 90 mg of Aronia melanocarpa berry extract powder for 24 weeks (*P* = 0.009) without providing an effect size [49]. For blueberry powder supplementations, Boespflug et al. reported marginally improved accuracy for the blueberry group in the 1-back condition (*P* = 0.08) for executive function assessment with a large effect size (Cohen’s *d* = 1.02) [46]; Miller et al. reported significantly fewer repetition errors in the California Verbal Learning test (*P* = 0.031) with a medium effect size (Cohen’s *d* = 0.50) and reduced switch cost on a task-switching test (*P* = 0.033) for executive function assessment across visits, relative to controls, whereas no effect on mood assessment using Geriatric Depression Scale (GDS) and the Profile of Mood States (POMS) was found [48]; Whyte et al. reported no effect on cognition and mood as assessed by the Positive and Negative Affect Schedule-NOW [61]. One blueberry concentrate study also reported improved executive function assessed by working memory (two back test) relative to the control (*P* = 0.05) [45].

For grape powder supplementation, Calapai et al. reported better attention (*P* < 0.001); language (*P* < 0.05); immediate memory (*P* < 0.0001); delayed memory (*P* < 0.0001) and MMSE score (*P* < 0.001) compared to the control without providing effect sizes [51]; Lee et al. also reported better attention/working memory under the domain of executive function, as measured with WAIS-III Digit Span within the intervention group (*P* = 0.04) without providing effect sizes, no effect on mood as assessed by Hamilton Rating Scale (HRS) was shown [52]. For grape juice supplementations, Lamport et al. reported better immediate spatial memory with a small effect size (Cohen’s *d* = 0.2) and driving performance (*P* < 0.05) with a small effect size (Cohen’s *d* = 0.4) compared to the control [43]; Kirkorian et al. reported attenuated cognitive error (5.03 vs 7.16 interference errors on recognition memory task) with a large effect size (Cohen’s *d* = 1.0) and no effect on mood as assessed by GDS [59]; Krikorian et al. reported improved Paired Associate Learning (PAL) (*P* = 0.009), Word List Recall (*P* = 0.04) with a medium effect size (Cohen’s *d* = 0.56) and trend for improving mood measured as reduced depressive symptoms (*P* = 0.08) [60].

For cherry juice supplementations, Chai et al. reported increased subjective memory in the domain of contentment with memory by 5%, reduced movement time by 4% and also reduced errors in episodic visual memory by 23% compared to control drink as assessed by PAL task without providing the effect size [41]. Kent et al. reported better memory and executive as assessed by verbal fluency (*P* = 0.014) with a large effect size (Cohen’s *d* = 1.04), short-term memory (*P* = 0.014) with a medium effect size (Cohen’s *d* = 0.79) and long-term memory (*P* ≤ 0.001) with a large effect size (Cohen’s *d* = 0.94) [50]. Morehen et al. found no significant changes in assessed sleep, fatigue or mood between pre- and post-intervention and between groups [58].

For cranberry juice supplementation, there is no effect (*P* = 0.123) on self-report memory [44]. For orange juice supplementation, there was improved global cognitive function and executive function (significant drink x visit interaction, *P* < 0.05) with no effect on Positive and Negative Affect Scale without providing an effect size [57].

### Meta-analyses

As shown in Table 1, for studies included in the meta-analysis, memory was assessed as either the number of correct responses or accuracy (%) in Immediate Word Recall, Delayed Word Recall or CVLT List Free Recall (California Verbal Learning Test); executive function was assessed as the total score, or the number of correct responses or arcsine transformation of the square root of the proportion of correct answers in Digit Symbol Substitution Test, Digit Span, 2-Back Task, Rapid Visual Information Processing (RVIP) or Number Cross-out Test; psychomotor speed was assessed as reaction time (RT, ms or s) of Trail Making Task, Reaction Time Test (RTI), Simple Reaction Time, 2-Back Task or RVIP. Studies incorporating grape juice, grape powder, blueberry juice, blueberry powder, cranberry juice and frozen blueberry constituting a berry group along with studies supplementing cherry juice were able to provide sufficient data to run meta-analysis. There was insufficient data for mood to be entered into meta-analysis, but the mood assessment results for individual studies are reported in Table 3.

**Fig. 3:**
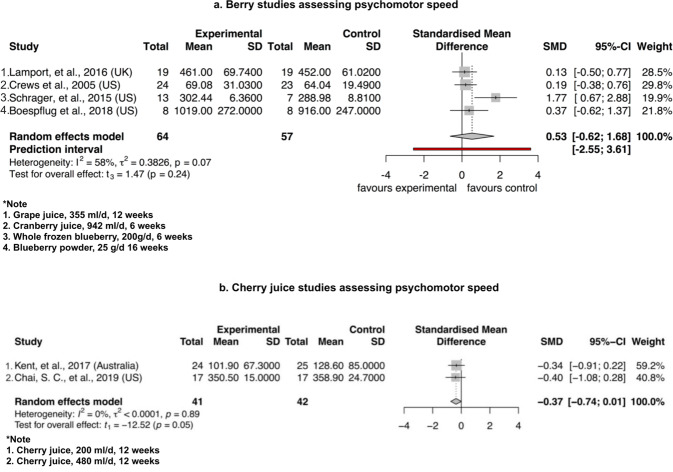
Forest plot of fruit studies assessing psychomotor speed. Forest plot of **a** berry studies and **b** cherry juice studies assessing psychomotor speed.

**Fig. 4:**
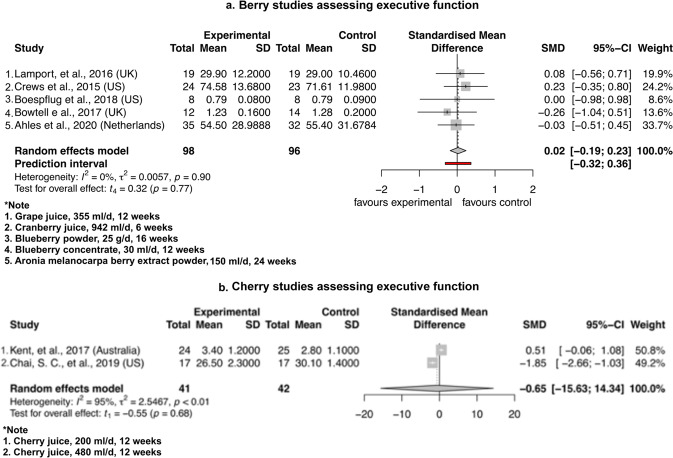
Forest plot of fruit studies assessing executive function. Forest plot of **a** berry studies and **b** cherry juice studies assessing executive function.

**Fig. 5:**
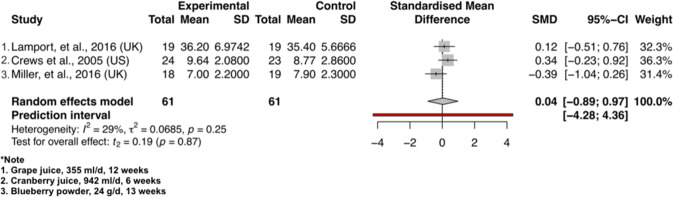
Forest plot of berry studies assessing memory.

**Fig. 6:**
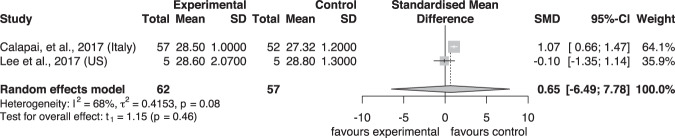
Forest plot of grape powder studies assessing MMSE. MMSE: Mini Mental State Examination.

Notably, cherry juice induced a borderline significance in improvement of psychomotor speed after the intervention compared to control (SMD = −0.37, 95% CI – 0.74 to 0.01, *P* = 0.05, t_1_ = −12.52) in Fig. 3. The berry group induced no significant difference for psychomotor speed between intervention and control group (SMD = 0.61, 95% CI −1.58 to 2.80, *P* = 0.36, t_4_ = 1.19) (Fig. 3). As shown in Fig. 4 for the assessment of executive function, the berry group induced no significant difference between intervention and control group (SMD = 0.02, 95% CI −0.19 to 0.23, *P* = 0.77, t_4_ = 0.32) and cherry juice induced no significant difference between the two groups (SMD = −0.65, 95% CI – 15.63 to 14.34, *P* = 0.68, t_1_ = −0.55). As shown in Fig. 5 for memory assessment, the berry group induced no significant difference between intervention and control groups (SMD = 0.04, 95% CI −0.89 to 0.97, *P* = 0.87, t_2_ = 0.19). Apart from the analysis assessing cognitive domains, two grape powder studies were able to provide MMSE (Mini Mental State Examination) data, but no significant difference was shown between intervention and control groups (SMD = 0.65, 95% CI – 6.49 to 7.78, *P* = 0.46, t_1_ = 1.15) (Fig. 6).

Significant heterogeneity among studies was observed in the cherry juice studies assessing executive function (*I*^*2*^ = 95%, *P* < 0.01) and berry group assessing psychomotor speed (*I*^*2*^ = 72%, *P* = 0.03). There was no change of heterogeneity in executive function and psychomotor speed assessment after the sensitivity analysis excluding studies applying physical activity adjustments and supplementing concentrate, the sensitivity analysis also suggested no effect of physical activity level and juice quality on the interventional effect (Table 4). Funnel plots and the egger’s test for the berry group showed an overall symmetric distribution of the berry interventions around the standard error for the investigated outcomes of executive function (Egger’s tests *P* = 0.24) and memory (Egger’s tests *P* = 0.28); asymmetric distributions were shown for the berry interventions investigating the effect on psychomotor speed (Egger’s tests *P* = 0.28); cherry interventions investigating the effect on executive function (Egger’s tests *P* = 0.35) and grape powder interventions investigating the effect on MMSE (Egger’s tests *P* = 0.24) (Supplemental Fig. 1). Trim and fill method was further implemented to adjust for publication bias (Supplemental Fig. 1).

**Table 4:** Sensitivity analysis.

	Berry group assessing memory				Cherry juice study assessing executive function				Cherry juice study assessing psychomotor speed			
	*N*	standardised mean difference (95% CI)	P for heterogeneity	P for overall effect	*N*	standardised mean difference (95% CI)	P for heterogeneity	P for overall effect	*N*	standardised mean difference (95% CI)	P for heterogeneity	P for overall effect
All studies	3	0.04 [−0.89; 0.97]	0.25	0.87	2	−0.65 [−15.63; 14.34]	<0.01	0.68	2	−0.37 [−0.74; 0.01]	0.89	0.05
Without adjusted studies^a^	2	0.24 [−1.15; 1.64]	0.62	0.27	1	0.51 [−0.06; 1.08]	NA	NA	1	−0.34 [−0.91; 0.22]	NA	NA
		berry group assessing executive function			cherry juice study assessing executive function				cherry juice study assessing psychomotor speed			
	*N*	standardised mean difference (95% CI)	P for heterogeneity	P for overall effect	*N*	standardised mean difference (95% CI)	P for heterogeneity	P for overall effect	*N*	standardised mean difference (95% CI)	P for heterogeneity	P for overall effect
All studies	5	0.02 [−0.19; 0.23]	0.9	0.77	2	−0.65 [−15.63; 14.34]	<0.01	0.68	2	−0.37 [−0.74; 0.01]	0.89	0.05
Without juice concentrate studies	4	0.07 [−0.13; 0.45]	0.93	0.34	1	0.51 [−0.06; 1.08]	NA	NA	1	−0.34 [−0.91; 0.22]	NA	NA

^a^Study’s statistical analysis has adjusted for participants’ physical activity.

## Discussion

### Principal findings

Our systematic review collected a range of frozen, freeze-dried, powdered and juiced fruit interventions, specifically berries (*n* = 12), reporting positive findings on cognition or mood. The largest portion of studies involved fruit juices (nine out of 16 studies) with grape juice (*n* = 3) and cherry juice (*n* = 3) being the most intensively studied supplementations. As demonstrated by the meta-analysis, two cherry juice studies including 41 participants receiving cherry juice with mean dosage of 340 ml/d for 12 weeks induced a borderline significant improvement in psychomotor speed. However, it is important to note that only two studies were included and one of those had a high risk of bias [41], which limits the impact of this finding.

Our meta-analyses suggested no differences between the intervention and control groups in any cognitive domains following berry or other fruit-based supplementations. No improvement was observed on any outcomes including executive function and memory, which does not support the notion that the consumption of specific fruit powders or other fruit juices will confer a cognitive-protective benefit. Overall, the individual interventions showing improvements in our systematic review still require further substantiation given that the meta-analysis only suggests that cherry juice may have cognition-protective potential.

### Scientific analysis of findings

The systematic review suggested the potential for whole blueberries, blueberry juice concentrate, blueberry powder, grape powder, grape juice, cherry juice, orange juice and cranberry juice supplements to improve cognitive health. Supplementing grape juice also showed potential to improve mood. However, from the meta-analysis, we only found a borderline significant improvement in psychomotor speed following chronic consumption of cherry juice. The participants of the cherry juices studies included here were older, healthy (> 60 years old) or have dementia. It’s worth noting that slower psychomotor speed has been found to be associated with increased risk of all-type dementia (hazard ratio [HR] 3.41, *P* < 0.0001), Alzheimer’s disease-type dementia (HR 3.18, *P* < 0.0001), Parkinson’s disease (HR 2.98, *P* = 0.04) and depressive symptoms (HR 1.53, *P* = 0.03) [65], and is therefore related to chronic mobility disorders and important for health wellbeing. Cherry juice has high content of flavonoids catechin, epicatechin, procyanidins and anthocyanins [66, 67] and the most recent evidence points towards potential benefits of supplementing flavonoids, ranging from 60 to 768 mg daily on attention, working memory, and psychomotor speed, but the study findings were not conclusive [68].

In our review, grape powder and juice interventions have shown improvement to cognition or mood for at least one of the cognitive aspects [43, 52, 59, 60, 62]. Studies supplementing blueberry powder, cherry juice and cranberry juice also have reported effect on cognitive or mood benefit [46, 48, 50, 64, 69, 70]. Polyphenol-rich fruit juice interventions that reported positive effect on cognitive health have provided anthocyanin levels ranging from 138 to 387 mg or 435 to 450 mg total polyphenol levels daily; fruit powder interventions with positive findings on cognition have provided either anthocyanin levels ranging from 12.5 to 460.8 mg or 70 mg total polyphenol levels daily. Small molecules, especially anthocyanins are the major class of polyphenols in berry and cherry fruit (approximately 92 mg/100 g) [41]. However, the anthocyanin profile in polyphenol-rich fruit is a factor explaining variability in the biological responses observed in dietary interventions with this fruit. For instance, blueberries contain primarily delphinidin, malvidin, and petunidin whereas raspberries and blackberries contain primarily cyanidin, pelargonidin and malvidin [71]. Although consumption of anthocyanins can be in the range of 200 mg/d [72], the bioavailability appears low as they are believed to be poorly absorbed and rapidly excreted [73]. The rate of polyphenol absorption from blueberries could be influenced by dose administered [74], and the matrix of the food source [75], and several studies have suggested that the rate of anthocyanin absorption is influenced by their chemical structure. The bioavailability of polyphenol metabolites will vary between individuals and is dependent on complex absorption, distribution, metabolism and excretion (ADME) mechanisms involving phase I and II metabolism of phenolic molecules [76]. Also, a systematic review has shown that the intake of fruit juice offered similar protection against cognitive decline to the intake of whole fruit [77] and thus a similar proportion of bioactive phytochemicals must remain in the processed products. Unfortunately, the current meta-analyses did not sufficiently support a role for fruits or other fruit forms to improve cognition and mood.

Although our meta-analysis lacks evidence to support improvements in mood by specific fruit interventions, another meta-analysis with 10 observational studies involving 227,852 participants suggested an inverse association of fruit (RR 0.83, 95% CI [0.77, 0.91]; *P* = 0.006) intake with risk of depression [78]. A previous systematic review has also assessed the association between cognitive benefits and fruit consumption but only included limited evidence from juice interventions, where improvements to memory in mildly cognitive-impaired adults after 12–16 weeks of consumption were illustrated [12]. The high levels of flavonoid metabolites (e.g. anthocyanidin) from flavonoid-rich fruit can transport through blood brain barrier into regions such as the hippocampus to impact on memory and learning [79]. In the pathogenesis of neurological conditions such as Parkinson’s disease, the mechanisms of action of flavonoids have been shown to counteract the damage induced by reactive oxygen species (ROS) and neuroinflammation, modulate synaptic signalling and increase cerebrovascular blood flow [77]. The (poly)phenol metabolites attenuated neuro-inflammatory processes *via* regulation of nuclear factor (NF)-κB translocation into the nucleus and modulation of IκBα levels by crossing the blood brain barrier endothelium and exerting beneficial effects in different neuronal systems (*e.g*. cell lines, primary cultures and in vitro three-dimensional human cell model) [80]. Therefore, cerebral metabolism of polyphenol and/or flavonoid molecules plays an important role in the preservation of cognitive function [81].

### Implications for health and future research

Currently, the majority of evidence in this area has included the association between intake of fruit combined with vegetable intake and cognitive function. However, the evidence on the association between specific fruit groups and/or forms of fruit with cognition is limited. Although insufficient data were entered for meta-analysis for each fruit subgroup, blueberry and grape powders providing 12.5–460.8 mg/d anthocyanin content were supplemented the most apart from fruit juice and could also be an effective method to increase overall fruit consumption and benefit cognition with moderate to large effect sizes reported [41, 44–48, 50–52, 57, 59–61]. Long-term investigations assessing the impact of whole blueberry intervention on cognition are also scarce and worth exploring, given that a large effect size was reported [47]. Thirteen out of 16 total RCTs in this review recruited older participants and only one study recruited young adults (18 years old athletes) without any improvements to cognition or mood shown. It could be due to that participants with relatively higher cognitive level at the baseline were unlikely to achieve higher cognitive response further following a dietary intervention due to ‘ceiling effects’ [82].

Only one intervention examined the effect of long-term supplementation with whole frozen fruit on cognition in this review, no fresh fruit was identified in the previous interventions, which could be due to the difficulty of storage and allocation during the intervention. Fresh whole fruit is generally how the fruit is consumed, which highlights a novel and necessary intervention in future studies. So far, research has mainly focused on fruit juice interventions, nevertheless, we should take free sugar reduction into account and the daily consumption of fruit juice should not exceed 150 ml per day as set out by Public Health England guidelines [83]. The sugar found in fruit juice is mainly classified as ‘free’ sugars, such as sucrose, whereas in whole fruit the sugars are classified as intrinsic. Increased dietary fructose following sucrose intake is reported to increase de novo lipogenesis and very-low-density lipoprotein levels, which has been shown to increase the risk of developing non-alcoholic fatty liver disease [84]. Therefore, any health promotion effects of increasing fruit intake by juice consumption should be made with caution. Validation of metabolites and biomarkers for cognitive impairment should be incorporated into future trials to help identify the potential mechanisms underlying any influence between fruit-based intake and cognitive health. Due to the impact of fruit processing and food matrix, cognitive research implementing fruit interventions should also consider controlling for factors likely to influence bioavailability in a chronic intervention (>1 week) [26].

### Strengths and limitations

To our knowledge, this is the first systematic review and meta-analysis to compare the impact of various forms of specific fruits in isolation from other food supplementation on cognition. Firstly, in addition to the comprehensive search of the literature in the topic, we also applied the newly developed Hartung-Knapp-Sidik-Jonkman method for modelling random effects in meta-analysis. Secondly, the interventions included in this review have assessed either general cognitive performance or specific cognitive domains using well-established cognitive tasks.

There are limitations to our review. Although our systematic review showed positive results in interventions supplementing with blueberry or grape, the small sample size and moderate to high risks of bias for studies quality that used the same cognitive task in the searched literature may partially explain the lack of support from our meta-analysis. It also should be noted that generally the cognitive tasks chosen in each intervention study are not uniform because of the polyfactorial nature of neurocognitive measures [85]. In the current review, not all the domain-specific tasks data could be entered into meta-analyses assessing the effect of the same type of fruit interventions on the specific cognitive domain, thus the effect size derived from the meta-analyses was inevitably under-estimated.

Study quality of 13 studies as assessed by risk of bias in this review presented with moderate to high risks in either randomisation process, intervention deviations, missing outcome data, and/or the measurement of the outcomes (Fig. 2). A crossover design is the most appropriate design when comparing a nutritional intervention against placebo since all participants serve as their own control and only three studies applied a crossover design [43, 57, 58]. Only one citrus intervention RCT supplementing orange juice was included, which led to limited exploration of the effect of citrus fruit intervention on cognition [43]. Due to the small number of studies in each pooled analysis, we were also unable to evaluate whether the effects can be influenced by participant characteristics (e.g. physical activity, sex) or to explore the high heterogeneity by subgroup analysis.

## Conclusion

This systematic review has identified berries with the most potential to benefit cognition, however, the meta-analyses only supported a borderline significant improvement to psychomotor speed by two small studies supplementing cherry juice with mean dosage of 340 ml/d for 12 weeks. To our knowledge, this is the first review of the impact on cognitive health following consumption of different varieties of fruit and different processed forms such as freeze-dried powdered fruit or fruit juice. Apart from fruit juice, promising results were also demonstrated among limited studies supplementing whole frozen or powdered forms of fruit (grape powder, frozen and powdered blueberries).

## Supplementary information

Supplemental Material

## Data Availability

The data that support the findings of this study are available from the corresponding author, JL, upon reasonable request.
